# Nitric Oxide Sustains Long-Term Skeletal Muscle Regeneration by Regulating Fate of Satellite Cells Via Signaling Pathways Requiring Vangl2 and Cyclic GMP

**DOI:** 10.1002/stem.783

**Published:** 2011-11-14

**Authors:** Roberta Buono, Chiara Vantaggiato, Viviana Pisa, Emanuele Azzoni, Maria Teresa Bassi, Silvia Brunelli, Clara Sciorati, Emilio Clementi

**Affiliations:** a Division of Regenerative Medicine, San Raffaele Scientific InstituteMilano, Italy; b Unit of Clinical Pharmacology, Consiglio Nazionale delle Ricerche Institute of Neuroscience, Department of Clinical Sciences L.Sacco, University Hospital “Luigi Sacco,” Università di MilanoMilan, Italy; c E. Medea Scientific InstituteBosisio Parini, Lecco, Italy; d Department of Experimental Medicine, University of Milano-BicoccaMonza, Italy

**Keywords:** Muscular dystrophy, Signal transduction, Self-renewal, Skeletal muscle

## Abstract

Satellite cells are myogenic precursors that proliferate, activate, and differentiate on muscle injury to sustain the regenerative capacity of adult skeletal muscle; in this process, they self-renew through the return to quiescence of the cycling progeny. This mechanism, while efficient in physiological conditions does not prevent exhaustion of satellite cells in pathologies such as muscular dystrophy where numerous rounds of damage occur. Here, we describe a key role of nitric oxide, an important signaling molecule in adult skeletal muscle, on satellite cells maintenance, studied ex vivo on isolated myofibers and in vivo using the α-sarcoglycan null mouse model of dystrophy and a cardiotoxin-induced model of repetitive damage. Nitric oxide stimulated satellite cells proliferation in a pathway dependent on cGMP generation. Furthermore, it increased the number of Pax7^+^/Myf5^−^ cells in a cGMP-independent pathway requiring enhanced expression of Vangl2, a member of the planar cell polarity pathway involved in the Wnt noncanonical pathway. The enhanced self-renewal ability of satellite cells induced by nitric oxide is sufficient to delay the reduction of the satellite cell pool during repetitive acute and chronic damages, favoring muscle regeneration; in the α-sarcoglycan null dystrophic mouse, it also slowed disease progression persistently. These results identify nitric oxide as a key messenger in satellite cells maintenance, expand the significance of the Vangl2-dependent Wnt noncanonical pathway in myogenesis, and indicate novel strategies to optimize nitric oxide-based therapies for muscular dystrophy. Stem Cells 2012; 30:197–209.

## INTRODUCTION

The ability of postnatal skeletal muscle to continuously regenerate its fibers is mostly dependent on satellite cells (SCs), myogenic precursors located under the basal lamina of myofibers, although other interstitial stem cells recently described may also play a role [1]. SC activate and differentiate on injury and are characterized by reversible quiescence and self-renewal capacities that are critical to sustain tissue regeneration during numerous rounds of damage [2–4]. The state of quiescence is characterized by the persistence of a G_0_ phase, the expression of several markers including M-Cadherin, Syndecans 3 and 4, CD34, α-7 integrin, SMC 2.6 [5–8], the expression of the paired-box protein Pax7, a regulator of cell survival and myogenic progression, and by the concomitant lack of expression of myogenic determination genes [9, 10]. On activation, SC proliferate and give rise to a population of myoblasts expressing myogenic regulatory factors which includes Myf5; these cells progressively downregulate Pax7 and, after multiple rounds of cell division, terminally differentiate to finally fuse and generate new fibers [11, 12]. A subset of SC maintains or re-expresses Pax7 and returns to quiescence by both symmetric and asymmetric divisions [13, 14]. This “reserve cell” pool is particularly relevant as it is the one accounting physiologically for muscle repair throughout life span. The importance to possess a SC renewable pool and thus an efficient muscle repair mechanism emerges clearly in muscular dystrophies, where genetic alterations in genes coding for structural muscle proteins lead to repeated and enhanced muscle damage during physiological activity. Although in initial phases of the disease the activity of SC may repair muscle damage, with time the myogenic precursor cell pool is progressively exhausted leading to a fibrotic and dysfunctional muscle [15].

Several factors involved in SC self-renewal have now been identified, including Myostatin/Smad3 [16–18], Angiopoietin1/Tie2 [19], Sprouty [20], Syndecan-3/Notch [21], Notch/Delta1, and Numb [4, 13, 22, 23]. Recent studies have also identified the key role of members of the canonical/β-catenin (β-Cat)-dependent and of the noncanonical Wnt pathways, the latter involving Wnt7a, its receptor Frizzled7 (Fzd7) and Vangl2, the mammalian homolog of the *Drosophila* Vangl2/Stbm [24–31]. Despite these studies, strategies to maintain SC renewal that can be exploited in a therapeutic perspective for muscular dystrophy are still lacking.

Here, we demonstrate that nitric oxide (NO), a key signaling molecule that controls adult skeletal muscle structure, bioenergetics, and function [32–34], and whose generation is altered in dystrophic muscles [35–38], maintains and regenerates the SC reserve pool. NO stimulates proliferation of SC and maintains the pool of Pax7^+^/Myf5^−^ SC following chronic and acute repetitive muscle injuries. NO stimulates proliferation of SC via cyclic GMP (cGMP) generation while the maintenance of the pool of Pax7^+^Myf5^−^ SC depends on the Vangl2-dependent Wnt noncanonical pathway. In addition, we identify in molsidomine, a NO releasing drug approved for use in humans, a very promising NO donating system endowed with great efficacy that may be successfully developed as a therapeutic for muscular dystrophies.

## MATERIALS AND METHODS

### Animals and Treatments

α-Sarcoglycan (α-SG) null mice, matched 129S2/SvpasClr control wild-type mice, C57BL/6J wild-type mice, and neuronal NO synthase (nNOS) null mice were housed in the pathogen-free facility at our Institution and treated in accordance with the European Community guidelines and with the approval of the Institutional Ethical Committee.

Standard diet (STD) or a diet containing 3 mg/kg of (1-ethoxy-*N*-(3-morpholino-5-oxadiazol-3-iumyl)methanimidate (molsidomine) was prepared based on the daily food intake measured for these animals [39]. No significant differences in food intake and weight gain were observed among the experimental groups. N^ω^nitro-L-arginine methylester (L-NAME, 1 mg/ml) was administered in drinking water.

In the experiments of cardiotoxin (CTX)-induced damage on wild-type mice (five animals/group for each experiment aged 6–8 weeks), drug treatments were started 1 week before CTX injection and continued until sacrifice. CTX (50 μl of a 10 μM solution) was injected using an insulin needle in both the *Tibialis Anterior* (TA) and quadriceps muscles. In parallel, two groups of mice (five animals per group) were injected with phosphate-buffered saline (PBS) as control. For the multiple damage experiments, CTX was injected three times every 5 days. The mice were sacrificed 10 days after injury to collect fibers from the *Extensor Digitorum Longus* muscle (EDL) or to isolate TA for histological analysis. When used, 5 bromo-2′-deoxyuridine (BrdU, 50 mg/g of body weight) was injected intraperitoneally, on day 5 after CTX damage, 2 hours before sacrifice.

α-SG null mice (15 animals per group) were treated starting at 1 month of age for up to 9 months. Mice were sacrificed at 5 and 9 months of age for histological and single fibers analysis [39].

For embryo studies, drug treatments of female α-SG null mice were started a week before and were maintained during the gestation period. The morning of plug detection was estimated as E0.5. Animals were sacrificed, and E15.5 embryos were removed for histological and Western blot analysis.

### Single Myofiber Isolation

Mice were killed by cervical dislocation, and EDL muscles were carefully dissected. Muscles were digested in 0.1% collagenase type V for 20 minutes and individual myofibers were dissociated by trituration using a Pasteur pipette [40].

For experiments in floating conditions, isolated myofibers were cultured for 24 hours in 6 or 12 wells coated with horse serum to prevent fiber attachment, at 37°C in 5% CO_2_ in a plating medium (Dulbecco's modified Eagle's medium [DMEM] containing 10% horse serum, 0.5% chick embryo extract, 100 U/ml penicillin, 100 μg/ml streptomycin, and 50 μg/ml gentamycin). The fibers were then switched to a proliferation medium (DMEM containing 20% fetal bovine serum, 10% horse serum, 2% chick embryo extract, 100 U/ml penicillin, 100 μg/ml streptomycin, and 50 μg/ml gentamycin) and incubated with the NO donors 3-morpholinosydnonimine (SIN-1) or 1-[*N*-(2-aminoethyl)-*N*-(2-ammonioethyl)amino]diazen-1-ium-1,2-diolate (DETA-NO), L-NAME, the guanylate cyclase inhibitor 1H-[1, 2, 4]oxadiazolo[4,3-a]quinoxalin-1-one (ODQ), 8Br-cGMP, Wnt7a, or vehicle. When used, BrdU (14 mg/ml) was added daily starting from the day of fibers isolation [29]. Results shown are averages of three to five reproducible independent experiments as indicated by the *n* number in the figure legends.

### Immunofluorescence on Single Fibers

Single fibers, freshly isolated or cultured in vitro, were fixed with 4% paraformaldehyde for 30 minutes at room temperature and incubated in PBS containing 0.1% Triton X-100 and 10% goat serum (GS) for 30 minutes before antibody incubations in PBS containing 1% GS overnight at 4°C. For BrdU experiments, the fibers were incubated in PBS containing 0.5% Triton X-100 and 10% GS for 30 minutes before primary antibodies incubation [29]. The secondary antibody incubation was for 1 hour at room temperature.

Nuclei were stained with 10 μM Hoechst 33342 for 5 minutes at room temperature, and single fibers were mounted using the Prolong Gold antifade reagent. Results shown are averages of three to five reproducible independent experiments as indicated by the *n* number in the figure legends.

### Real-Time Polymerase Chain Reaction

Analyses were carried out on single fibers obtained from CTX-injected mice 10 days after damage or from 9-month-old α-SG null mice. RNA was prepared from freshly isolated single fibers using Trizol, and 1 μg of RNA per sample was reverse transcribed into cDNA using the QuantiTect Reverse Transcription kit according to the manufacturer's instructions. The expression levels of Myf5, Pax7, Wnt7a, Fzd7, Vangl2, and β-Cat were analyzed by quantitative real-time polymerase chain reaction (PCR) on an ABI PRISM 7900HT Fast Real-Time PCR Systems using specific gene expression assays. β2 Microglobulin and glyceraldehyde-3-phosphate dehydrogenase were used for normalization with similar results. Untreated fibers were used as endogenous reference. Data were analyzed using the δ-delta-Ct method. Results shown are averages of three to five reproducible independent experiments as indicated by the *n* number in the figure legends.

### Small Interfering RNAs' Transfections

Silencing of Vangl2 was obtained using a small interfering RNA (siRNA) duplex (ID s96801 and s96802) at the final concentration of 10 nM. The specificity was determined by the analysis of Vangl2 expression levels in myofibers transiently transfected with the specific siRNA duplex or with the scrambled control sequence by quantitative real-time PCR [25]. Transfections were carried out 16 hours after dissection in proliferation medium using Lipofectamine 2000. Transfection efficiency was monitored using a Cy3-labeled siRNA. RNA extraction or immunofluorescence analyses were performed 96 or 42 hours later. Results shown are averages of three to four reproducible independent experiments as indicated by the *n* number in the figure legends.

### Analysis of Biochemical and Functional Parameters in Dystrophic Mice

Serum creatine phosphokinase (CK) levels were measured in blood samples obtained by tail vein withdrawal exactly as described [39]. Functional muscle activity was measured using the running wheel, to assess free locomotor activity, and the exhaustion treadmill, to assess resistance to fatigue carried out exactly as described [39]. Averages of results obtained in 15 animals are shown in the figures.

### Immunoflurescence and Histology on Tissue Sections

Animals were sacrificed by cervical dislocation, and TA (CTX-injected mice) or diaphragm muscles (α-SG null mice) were dissected and immediately frozen in liquid N_2_-cooled isopentane [39]. For histological analysis, serial muscle sections were obtained and stained in hematoxylin and eosin (H&E) following standard procedures. Necrotic cells were identified by hypereosinophilia, thinning and waviness, and presence of many nuclei [41] while regenerating fibers were distinguished based on the central localization of their nuclei. Pax7^+^ cells were counted by immunofluorescence staining of muscle sections using anti-Laminin and anti-Pax7 antibodies exactly as described [42].

In the case of embryo experiments, samples were treated and cryosections were prepared as described [43]. All sections were taken on the sagittal plane and the depth of cut was established at the midline of the embryo and by using the Kaufman's atlas of embryology. Matched sections from at least three separate embryos were used for all analyses. Sections were mounted using the Prolong Gold antifade reagent. Averages of results obtained in 15 animals are shown in the figures.

### Sodium Dodecylsulfate Polyacrylamide Gel Electrophoresis and Western Blotting

Myogenic precursor cells isolated from leg adult muscles [44] or decapitated and eviscerated mouse embryos [43] were lysed and analyzed by sodium-dodecylsulfate electrophoresis exactly as described [45]. Averages of results obtained in 15 animals independently examined are shown.

### Statistical Analysis

Results are expressed as the means ± SEM. For the parameters with a normal distribution, the Student's *t* test was used, whereas the Mann-Whitney *U* test was used for parameters with a non-normal distribution. A *p* value <.05 was considered as statistically significant.

## RESULTS

### Nitric Oxide Affects Satellite Cells Fate

To study the effect of NO on SC fate, we analyzed Pax7 and Myf5 cell numbers on single myofibers isolated from EDL muscle and cultured in floating conditions in the presence or absence of the NO donor SIN-1, the NOS inhibitor L-NAME, or vehicle. These experimental conditions maintain SC anatomical location and allow their physiological transition across the activation and self-renewal phases [4]. Single myofibers isolated from age-matched nNOS^−/−^ mice were analyzed in parallel. In nonadherent culture conditions, myogenic precursor cells became activated immediately after isolation and underwent their first division within 36–42 hours, generating cell doublets that have clonal origin (Supporting Information Fig. 1A), as already described [22]. Pax7 and Myf5 expression was evaluated after 96 hours of culture, that is, at a stage in which Pax7^+^/Myf5^−^ and Pax7^−^/Myf5^+^ cells are considered self-renewing or committed to final differentiation into mature myoblasts, respectively [14, 19]. In our experimental conditions, all Myf5^+^ cells were also MyoD^+^ and about 90% ± 3.5% of Myf5^+^ cells expressed Myogenin, a late differentiation factor [11] (Supporting Information Fig. 1B).

**Figure 1:**
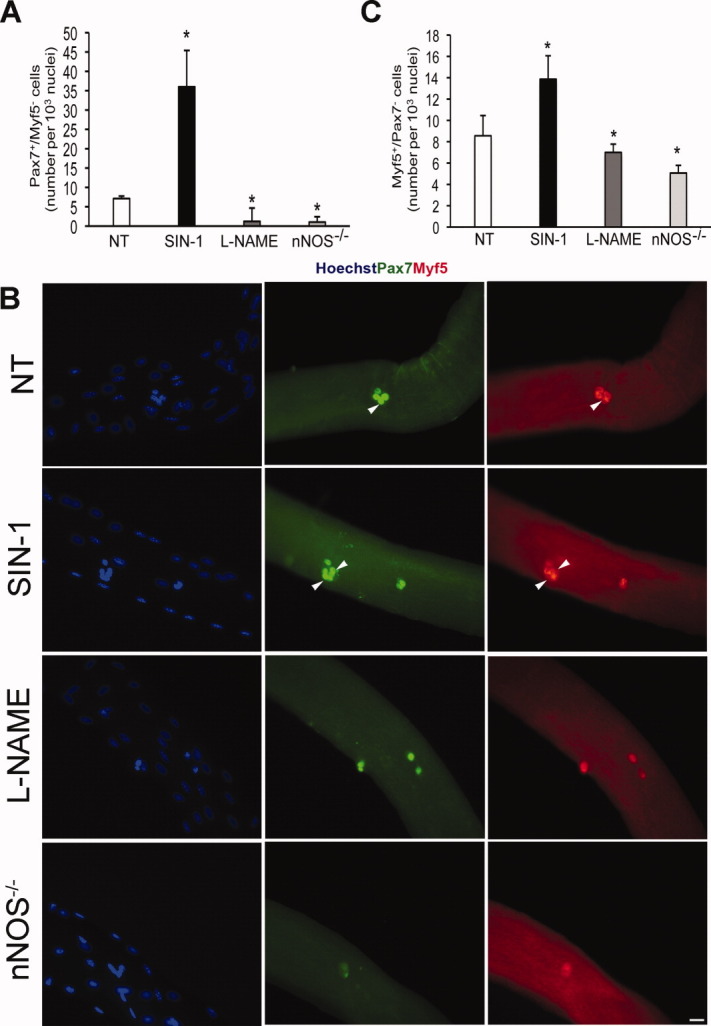
Nitric oxide (NO) increases the number of satellite cells in ex vivo isolated myofibers. (**A**): Number of Pax7^+^/Myf5^−^ cells in single fibers cultured in floating conditions. Fibers were obtained from wild-type mice and treated for 96 hours with the NO donor SIN-1 (3 μM), the NOS inhibitor L-NAME (3 mM), or vehicle (NT), or obtained from nNOS^−/−^ mice. Data are expressed as mean ± SEM and normalized for fiber nuclei number. Results are from at least 50 fibers for each experiment, *n* = 4; *, *p* ≤ .05 versus NT. Representative images of the results reported in (A) are shown in (**B**). Pax7^+^ cells are green, Myf5^+^ are red. Blue staining shows nuclei. Scale bar = 25 μm. (**C**): Number of Pax7^−^/Myf5^+^ in single fibers cultured in floating conditions. Data are expressed as mean ± SEM after normalization for fiber nuclei number. Results are from at least 50 fibers for each experiment, *n* = 4; *, *p* ≤ .05 versus NT. Abbreviations: L-NAME, N^ω^nitro-L-arginine methylester; NT, untreated; nNOS, nitric oxide synthase; SIN-1, 3-morpholinosydnonimine.

SIN-1 increased, while L-NAME decreased the number of Pax7^+^/Myf5^−^ cells. Consistently, the number of Pax7^+^/Myf5^−^ cells in single myofibers obtained from nNOS^−/−^ mice was reduced (Fig. 1A and 1B). The effects of SIN-1 and L-NAME were concentration dependent (Supporting Information Fig. 1C).

NO donation or removal also affected the number of Pax7^−^/Myf5^+^ cells that was increased by treatment with the NO donors and reduced by L-NAME or in nNOS^−/−^ mice. Of importance, such an effect was significantly less evident than the one observed on Pax7^+^/Myf5^−^ SC (1.4-fold vs. 14-fold increase with SIN-1, Fig. 1C), indicating that NO increased primarily the number of Pax7^−^/Myf5^+^ SC. Results similar to those yielded by SIN-1 were obtained using a chemically unrelated NO donor, DETA-NO (Supporting Information Fig. 1D), highlighting the dependence of the observed effects on NO.

### NO Increases the Number of Satellite Cells after Repetitive Cardiotoxin-Induced Muscle Damage and Favors Muscle Regeneration

After CTX injection, SC increased in number within 2–5 days (Supporting Information Fig. 2A). Approximately 10 days after, SC reappeared in a sublaminar position typical of quiescent cells [25] (Supporting Information Fig. 2A). At this stage, the formation of centronucleated-regenerating fibers was clearly observed (Supporting Information Fig. 2B).

**Figure 2:**
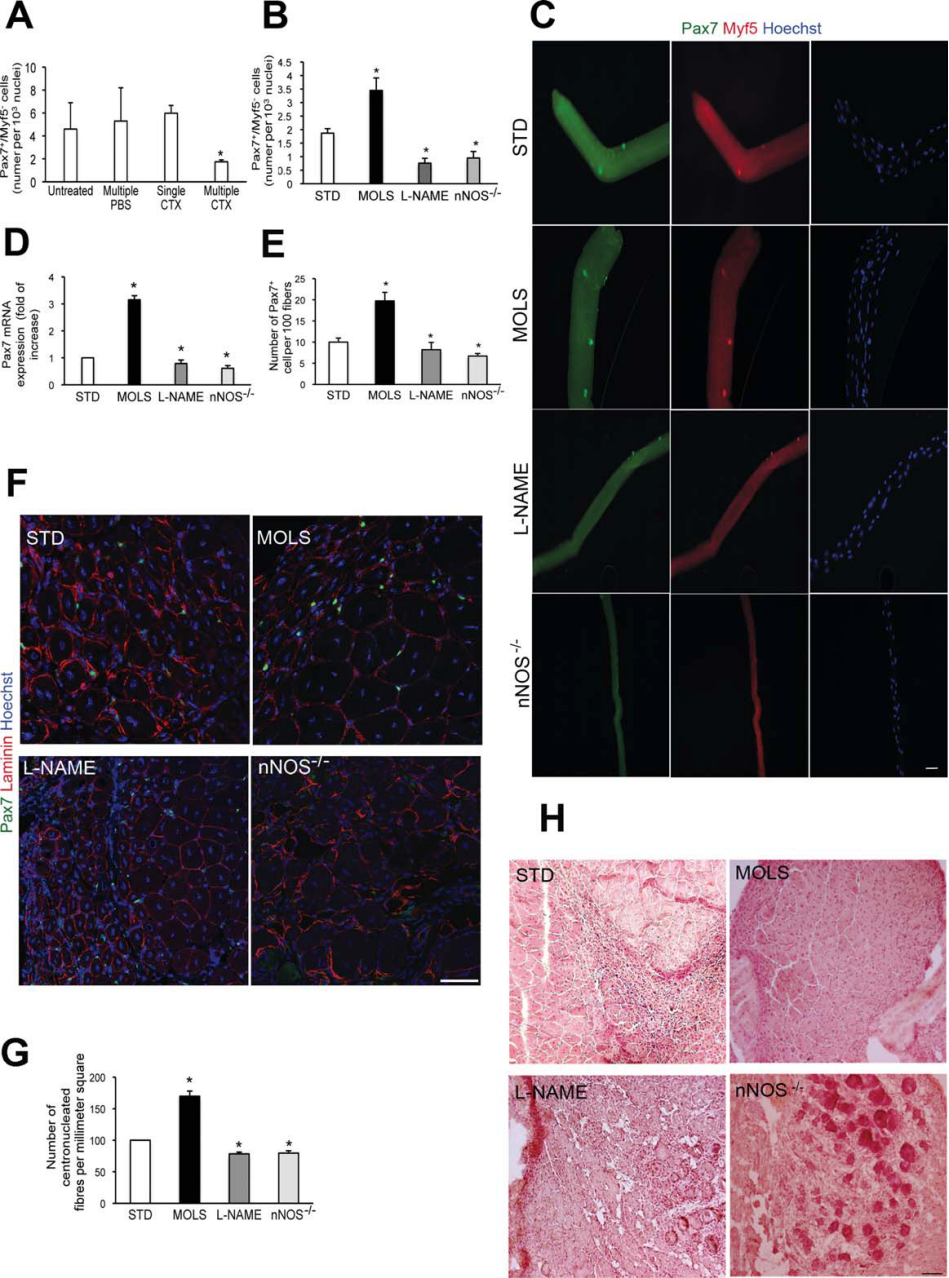
Nitric oxide (NO) increases the satellite cell pool after repeated CTX injections in vivo. (**A**): Number of Pax7^+^/Myf5^−^ cells in single fibers isolated 10 days after single or three (multiple) CTX injections. Data are expressed as mean ± SEM and normalized for fiber nuclei number. At least 50 fibers were analyzed for each experiment, *n* = 3; *, *p* ≤ .05 versus single damage. The number of Pax7^+^/Myf5^−^ cells counted in fibers isolated prior to CTX injection (untreated) or in mice receiving three (multiple) injections of PBS as control are also shown. (**B**): Number of Pax7^+^/Myf5^−^ cells counted in single fibers isolated from *Extensor Digitorum Longus* muscle (EDL) muscle after multiple injections of CTX. Fibers were either obtained from wild-type mice that received STD or a diet containing the NO donor MOLS (3 mg/kg) or with L-NAME (1 mg/ml) in drinking water, or isolated from nNOS^−/−^ mice. Data are expressed as mean ± SEM (≥100 fibers for each experiment, *n* = 3) and normalized for fiber nuclei number; *, *p* ≤ .05 versus STD. (**C**): Representative images of data shown in (B). Pax7^+^ cells are green, Myf5^+^ cells are red. Blue staining shows nuclei staining by Hoechst. Scale bar = 25 μm. (**D**): Real-time PCR analysis of Pax7 expression in myofibers isolated after CTX injection. Results are from three independent experiments and are reported as fold increases over STD, set arbitrarily at 1; *, *p* ≤ .05 versus STD. (**E**): Number of Pax7^+^ satellite cells counted in sections of isolated *Tibialis Anterior* (TA) muscles. Data are expressed as mean ± SEM and normalized for fiber number, *n* = 5; *, *p* ≤ .05 versus STD. (**F**): Representative images of Pax7-stained sections. Pax7 is green while laminin is red. Nuclei are blue. Scale bar = 75 μm. (**G**): Number of centronucleated fibers counted in hematoxylin and eosin (H&E) sections from TA muscles. Results are expressed as mean ± SEM and reported as fold increases over STD, set arbitrarily at 100, *n* = 5; *, *p* ≤ .05. (**H**): Representative images of H&E sections. Scale bar = 100 μm. Abbreviations: CTX, cardiotoxin; L-NAME, N^ω^nitro-L-arginine methylester; MOLS, molsidomine; nNOS, nitric oxide synthase; PBS, phosphate-buffered saline; PCR, polymerase chain reaction; STD, standard diet.

We used this model to evaluate the effect of NO on myogenic precursor cells fate in vivo and its importance for muscle regeneration. We investigated the effect of a pharmacological treatment with the NO donor molsidomine, which is converted in vivo to its active metabolite SIN-1, or with L-NAME. Animals received three injections of CTX or vehicle (PBS) at 5-day intervals. nNOS^−/−^ mice treated or not with CTX were investigated in parallel. Repeated, short-haul injury induced severe damage and reduced the number of SC due to the repeated cycles of damage and proliferation (Fig. 2A). The effect of NO was evaluated 10 days after CTX treatment by assessing single myofibers obtained from EDL, histological sections of TA, and myogenic cells isolated from hind limb muscles.

Single fibers freshly isolated after triple challenge with CTX from EDL of mice treated with L-NAME or from nNOS^−/−^ mice exhibited a significantly reduced number of Pax7^+^/Myf5^−^ cells compared with control animals. By contrast, molsidomine increased the number of Pax7^+^/Myf5^−^ cells in myofibers (Fig. 2A, 2B, 2C). Real-time PCR analyses of Pax7 mRNA expression in single myofibers confirmed these results (Fig. 2D). Consistent with the results reported in Figure 1, also in this case the number of Pax7^+^/Myf5^−^ cells was increased by NO significantly more than the number of Pax7^−^/Myf5^+^ cells (Supporting Information Fig. 2C, 2D).

In the histological sections, immunostaining revealed that the number of Pax7^+^ cells was increased in molsidomine-treated animals compared with the control and reduced in animals that received L-NAME as well as in nNOS^−/−^ mice (Fig. 2E, 2F). Consistent with this observation, molsidomine enhanced whereas L-NAME or nNOS ablation reduced the number of centronucleated fibers, indicating that NO stimulates regeneration (Fig. 2G and 2H).

Analysis of the number of myogenic cells residing in the G_0_ versus G_2_/M phases of cell cycle at day 10 after CTX damage confirmed the above results. Myogenic cells in G_0_, analyzed after exclusion of cells in the G_1_ phase by pyronin staining [19], express Pax7 at significant levels, consistent with their nature of quiescent myogenic precursors (Supporting Information Fig. 2E, 2F). Treatment with molsidomine increased the number of cells in the G_0_ phase after 10 days of damage whereas L-NAME dramatically reduced it with respect to animals receiving STD (Supporting Information Fig. 2F, 2G). Cells isolated from nNOS^−/−^ mice behaved as those treated with L-NAME (Supporting Information Fig. 2F, 2G).

These data indicate that NO, both endogenous and exogenously administered, increases the pool of myogenic precursor cell in vivo, delaying its reduction during repetitive CTX damage and sustaining regeneration of skeletal muscle.

### NO Stimulates Proliferation of Satellite Cells Via cGMP

To investigate how NO increases the number of SC, we analyzed its effect on SC proliferation measuring BrdU incorporation in fibers cultured in floating conditions in the presence or absence of SIN-1 or L-NAME, or in fibers isolated from molsidomine- or L-NAME-treated animals.

**Figure 3:**
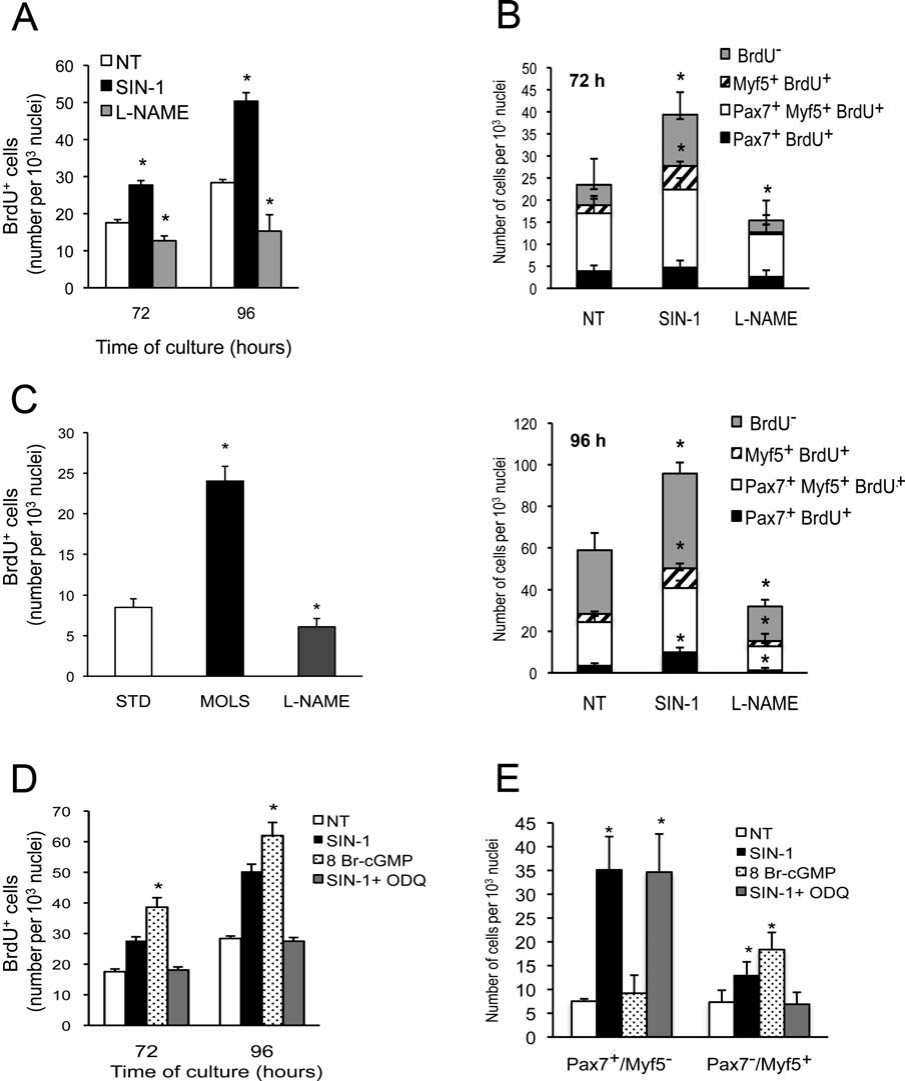
Nitric oxide (NO) stimulates satellite cells proliferation via cyclic GMP (cGMP). (**A**): Number of 5 bromo-2′-deoxyuridine (BrdU^+^) cells measured in single fibers cultured in floating conditions for 72 or 96 hours and treated with the NO donor SIN-1 or the NOS inhibitor L-NAME or vehicle (NT). Data are expressed as mean ± SEM and normalized on fiber nuclei number. Results from at least 50 fibers for each experiment, *n* = 4; *, *p* ≤ .05 versus NT. (**B**): Number of cells expressing Pax7 and/or Myf5 and/or labeled by BrdU, as indicated in the caption, in single fibers cultured in floating conditions for 72 (upper) or 96 (lower) hours. Data are expressed as mean ± SEM and normalized on fiber nuclei number. Results from at least 50 fibers for each experiment, *n* = 4; *, *p* ≤ .05 versus NT. (**C**): Number of BrdU^+^ cells in single fibers isolated from EDL muscle of wild-type mice that received STD or a diet containing MOLS or with L-NAME in drinking water. Data are expressed as mean ± SEM (≥100 fibers for each experiment, *n* = 3) and normalized on fiber nuclei number; *, *p* ≤ .05 versus STD. (**D**): Number of BrdU^+^ cells measured in single fibers cultured in floating conditions for 72 or 96 hours and treated with SIN-1 or the cell-permeable cGMP analog 8-Br cGMP (0.5 mM), SIN1 plus the inhibitor of the guanylate cyclase 1H-[1, 2, 4]oxadiazolo[4,3-a]quinoxalin-1-one (3 μM) or vehicle (NT). Data are expressed as mean ± SEM and normalized on fiber nuclei number. Results from at least 30 fibers for each experiment, *n* = 4; *, *p* ≤ .05 versus NT. (**E**): Number of Pax7^+^/Myf5^−^ and Pax7^−^/Myf5^+^ SC measured in single fibers cultured in floating conditions for 96 hours. Data are expressed as mean ± SEM and normalized on fiber nuclei number. Results are from at least 30 fibers for each experiment, *n* = 4; *, *p* ≤ .05 versus NT. Abbreviations: L-NAME, N^ω^nitro-L-arginine methylester; MOLS, molsidomine; NT, untreated; nNOS, nitric oxide synthase; SIN1, 3-morpholinosydnonimine;STD, standard diet; siRNA, small interfering RNA.

In isolated fibers treated ex vivo, SIN-1 increased while L-NAME reduced the number of BrdU^+^ SC after 72 and 96 hours of culture (Fig. 3A). Interestingly, more susceptible to the mitogenic effect of NO were the Pax7^−^/Myf5^+^ differentiation-determined SC (Fig. 3B), whereas the number of Pax7^+^/Myf5^−^ SC was not affected. Molsidomine significantly increased while L-NAME reduced the number of BrdU^+^ fibers with respect to fibers isolated from STD-treated animals (Fig. 3C).

We investigated the cGMP dependency of the mitogenic effect of NO by culturing single fibers in floating conditions in the presence or absence of the cell permeable analog of cGMP, 8-Br cGMP. 8-Br cGMP increased proliferation of SC similarly to what was observed with SIN-1 (Fig. 3D). The mitogenic effect of 8-Br cGMP was on the Pax7^−^/Myf5^+^ SC whereas it did not affect Pax7^+^/Myf5^−^ SC (Fig. 3E). Consistently, inhibition of cGMP generation by the guanylate cyclase inhibitor ODQ reverted the effect of SIN-1 only on Pax7^−^/Myf5^+^ SC (Fig. 3D, 3E). These results indicate that stimulation of proliferation by NO accounts for an increase in committed SC, an event already known to occur in other cell types through well-defined pathways [46, 47]; they also indicate that other mechanisms, independent of cGMP, are responsible for the NO-dependent increase in Pax7^+^/Myf5^−^ self-renewing cells.

### NO Regulates Vangl2 Expression and Enhances the Number of Pax7^+^/Myf5^−^ Satellite Cells via a cGMP-independent Mechanism

**Figure 4:**
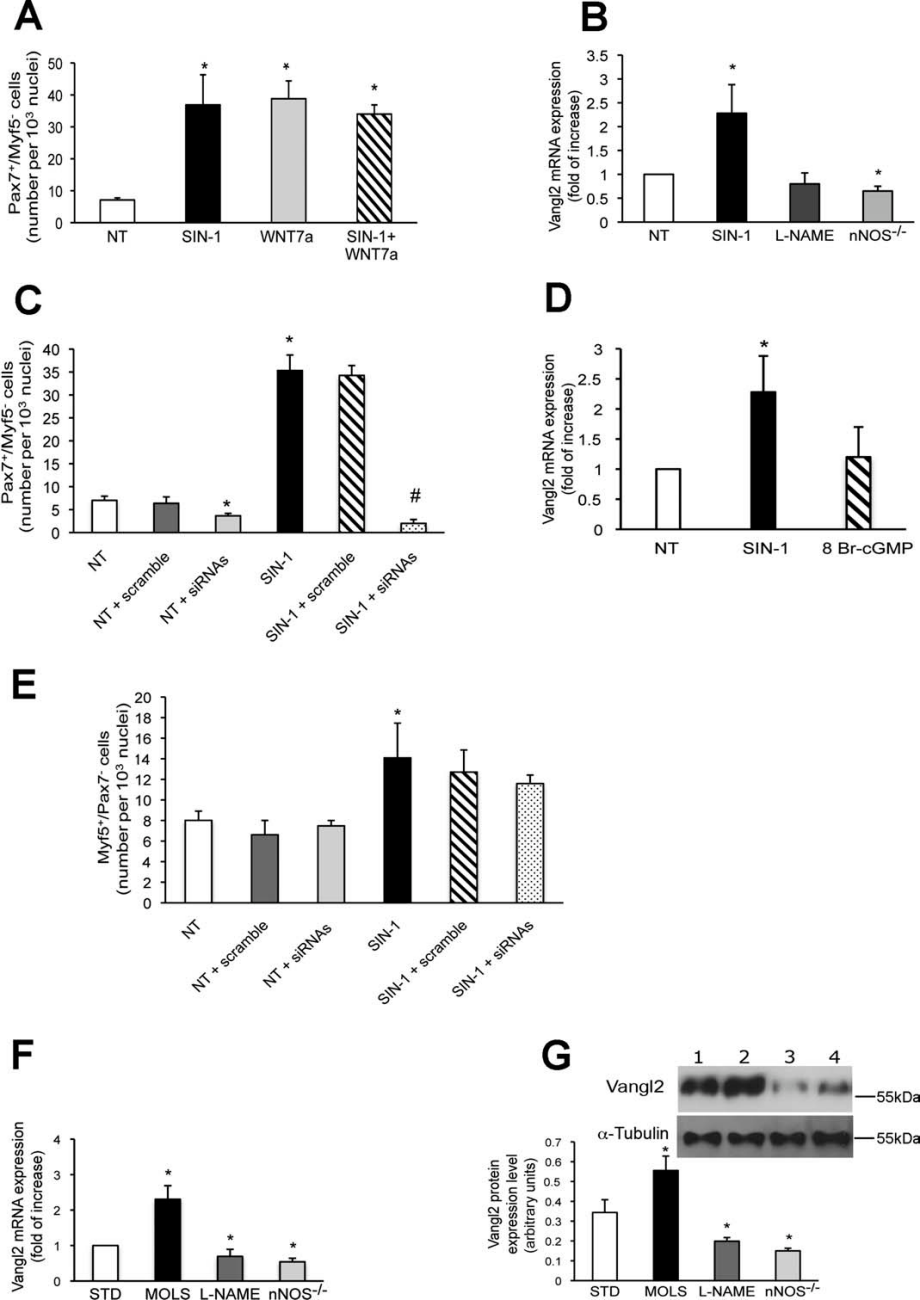
Vangl2 mediates the effect of nitric oxide (NO) on regulation of Pax7^+^/Myf5^−^ satellite cell numbers. (**A**): Number of Pax7^+^/Myf5^−^ in single fibers cultured in floating conditions and treated with SIN-1 or Wnt7a (25 ng/ml) alone or in combination. Vehicle-treated fibers are shown for comparison (NT). Data are expressed as mean ± SEM and normalized on fiber nuclei number. At least 50 fibers for each experiment, *n* = 4, were analyzed, *, *p* ≤ .05 versus NT. (**B**): Real-time polymerase chain reaction (PCR) analysis of Vangl2 expression in single fibers from wild-type or from nNOS^−/−^ mice, cultured in floating conditions with SIN-1, L-NAME, or vehicle (NT). Results are expressed as fold changes over the values in NT set arbitrarily at 1, *n* = 3, *, *p* ≤ .05 versus NT. (**C**): Number of Pax7^+^/Myf5^−^ SC in single fibers treated with SIN-1 or vehicle (NT) and transfected with scramble sequence or with the Vangl2 siRNAs (siRNA). Data are expressed as mean ± SEM and normalized on fiber nuclei number. Number of fibers analyzed was ≥50 for each treatment, *n* = 3, *, *p* ≤ .05 versus NT; #, *p* ≤ .05 versus scramble-transfected SIN-1 treated fibers. (**D**): Real-time PCR analysis of Vangl2 expression in single fibers cultured in floating conditions and treated with SIN-1, 8-Br cGMP, or vehicle (NT). Results are expressed as fold changes over the values in NT set arbitrarily at 1, *n* = 3, *, *p* ≤ .05 versus NT. (**E**): Number of Pax7^−^/Myf5^+^ SC in single fibers. Data are expressed as mean ± SEM and normalized on fiber nuclei number. The number of fibers analyzed was ≥50 for each treatment, *n* = 3, *, *p* ≤ .05 versus NT. (**F**): Real-time PCR analysis of Vangl2 expression in myofibers isolated after CTX injection. Fibers were from wild-type mice that received STD or a diet containing the NO donor MOLS or with L-NAME in drinking water, or isolated from nNOS^−/−^ mice. Results are expressed as fold changes over the values in STD set arbitrarily at 1, *n* = 3, *, *p* ≤ .05 versus STD. (**G**): Western blotting data showing expression of Vangl2 protein and α-tubulin, used as loading control, in lysates of myoblasts isolated from limb muscles, *n* = 3. *, *p* ≤ .05 versus STD. Representative images are shown in the inset (lane 1: STD, lane 2: MOLS, lane 3: L-NAME, lane 4: nNOS^−/^). Abbreviations: NT, untreated; L-NAME, N^ω^nitro-L-arginine methylester; MOLS, molsidomine; nNOS, nitric oxide synthase; siRNA, small interfering RNA; SIN-1, 3-morpholinosydnonimine; STD, standard diet.

The Wnt7a/Frzd7 signaling is specifically involved in symmetric expansion of the quiescent myogenic precursor cells pool [25]. In single fiber culture, SIN-1 and exogenously added Wnt7a on SC increased the number of Pax7^+^/Myf5^−^ cells in similar ways and no additive effects were observed by their coadministration (Fig. 4A). Treatment with SIN-1 increased the expression of Vangl2, assessed by real-time PCR experiments on RNA isolated from single fibers maintained in floating conditions (Fig. 4B). By contrast, no significant modifications were induced by SIN-1 in the expression levels of Wnt7a, Frzd7, or β-Cat (Supporting Information Fig. 3A).

To verify if the effect of NO on the number of Pax7^+^/Myf5^−^ SC required Vangl2 expression, we used a Vangl2-specific siRNAs to transfect single myofibers isolated from EDL, grown in floating condition for 96 hours and stimulated with SIN-1. Silencing efficiency was about 80% (Supporting Information Fig. 3B). Silencing of Vangl2 reduced the number of Pax7^+^/Myf5^−^ self-renewing cells in untreated fibers as previously described [25]; more importantly, it almost completely abolished the effect of SIN-1 (Fig. 4C). Of importance, 8-Br cGMP did not affect Vangl2 expression, demonstrating that the action of NO on Vangl2 is independent of cGMP generation (Fig. 4D). In the same conditions, the cGMP-dependent increase in the number of SC was not influenced by Vangl2 siRNAs transfection, further confirming the existence of two separate actions of NO on SC (Fig. 4E).

We investigated whether NO signaling via Vangl2 operated also in vivo. To this end, control animals receiving a STD, nNOS^−/−^ mice, and animals treated with L-NAME or molsidomine were subjected to CTX damage. We then analyzed the expression levels of Vangl2 by real-time PCR on single fibers freshly isolated from EDL as well as protein expression levels by Western blotting on myoblasts isolated from limb muscles. Vangl2 levels were significantly increased over control in fibers isolated from animals that received molsidomine. Consistently, the expression of Vangl2 mRNA and protein was reduced both in fibers obtained from L-NAME-treated animals and in nNOS^−/−^ mice (Fig. 4F, 4G). No changes were observed in the expression levels of Wnt7a and β-Cat, while Frzd7 levels were increased by NO treatment and reduced by NO ablation (Supporting Information Fig. 3C and not shown).

### Molsidomine Maintains the Satellite Cell Pool and Has Therapeutic Efficacy in Dystrophic Mice

**Figure 5:**
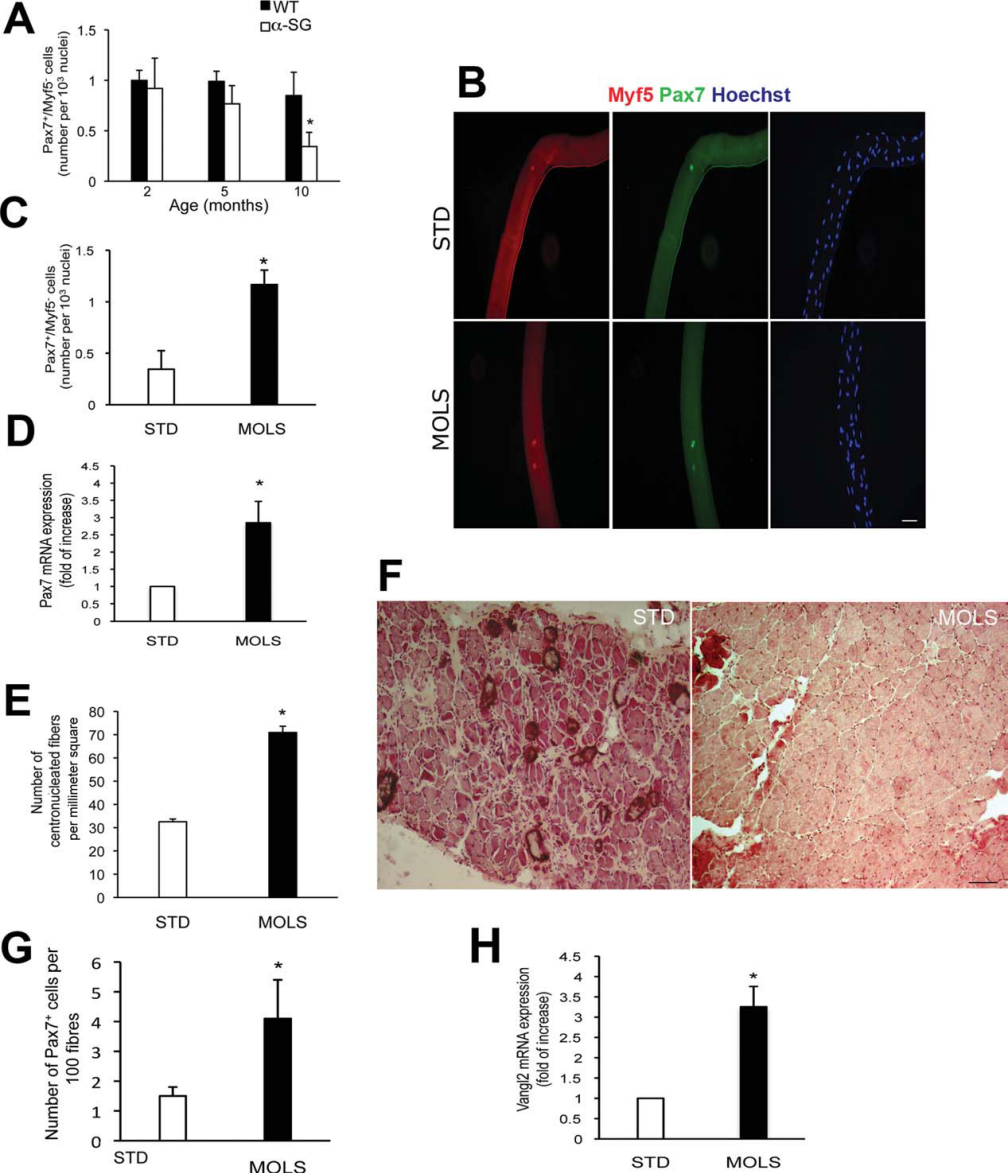
MOLS delays the reduction of the satellite cell pool in dystrophic mice. (**A**): Number of Pax7^+^/Myf5^−^ cells counted in single fibers isolated from α-SG null mice at 2, 5, or 10 months of age, compared with wild-type mice. Data are expressed as mean ± SEM and normalized on fiber nuclei number. Fiber number was ≥100, *n* = 3, *, *p* ≤ .05 versus 5 months. (**B, C**): Representative images and number of Pax7^+^/Myf5^−^ cells counted in single fibers isolated from α-SG null mice that received STD or treatment with MOLS. Data are expressed as mean ± SEM and normalized on fiber nuclei number. Fiber number was ≥100, *n* = 3, *, *p* ≤ .05 versus STD. Images in (B) show the staining for Pax7 (green), Myf5^+^ (red), and nuclei (Hoechst, blue). Scale bar = 75 μm. (**D**): Real-time polymerase chain reaction (PCR) analysis of Pax7 expression in myofibers isolated from α-SG null mice treated with STD or MOLS. Results are expressed as fold changes over the values in STD set arbitrarily at 1, *n* = 3, *, *p* ≤ .05 versus STD. (**E**): Number of centronucleated fibers counted in hematoxylin and eosin sections (representative images in **F**) and number of Pax7^+^ SC (**G**) in isolated diaphragm muscles of α-SG null mice. Data are expressed as mean ± SEM and normalized on the section area, *n* = 15. *, *p* ≤ .05 versus STD. Scale bar = 100 μM. (**H**): Real-time PCR analysis of Vangl2 expression in fibers isolated from α-SG null mice treated with STD or MOLS. Results are expressed as fold changes over the values in STD set arbitrarily at 1, *n* = 3, *, *p* ≤ .05 versus STD. Abbreviations: MOLS, molsidomine; α-SG, α-sarcoglycan; STD, standard diet; WT, wild type.

To examine the effects of NO on SC in muscular dystrophy, we relied on the α-SG null mice as it has a severe dystrophic phenotype and is a good model to assess the long-term efficacy of drugs active on muscular dystrophies [48]. In addition, it displays a severe depletion of the myogenic SC over time [39]. These mice are also characterized by a functionally inactive nNOS [37]. Animals were treated with STD or molsidomine from 1 month after weaning onward. The effect of the treatment was evaluated at 5 and 9 months of age, that is, when histological signs of muscle damage in untreated mice are clearly visible and muscle functional activity greatly impaired [39, 48, 49]. The number of Pax7^+^/Myf5^−^ SC in single fibers freshly isolated from EDL muscle of animals receiving STD progressively decreased with age compared with aged-matched animals (Fig. 5A). Analysis of fibers isolated from molsidomine-treated animals at 9 months of age showed a significantly higher number of Pax7^+^/Myf5^−^ SC (Fig. 5B, 5C) and real-time PCR analysis of Pax7 expression in isolated single fibers confirmed these results (Fig. 5D). The number of Pax7^−^/Myf5^+^-activated SC and Myf5 expression levels were also increased by molsidomine treatment (Supporting Information Fig. 4A, 4B).

Consistent with these observations, molsidomine increased the number of the centronucleated-regenerating fibers measured in diaphragm muscles (Fig. 5E, 5F) as well as the number of Pax7^+^ cells (Fig. 5G). In parallel, NO enhanced the expression of Vangl2 and Frzd7, with no changes in Wnt7a or β-Cat levels (Fig. 5H and Supporting Information Fig. 4C).

**Figure 6:**
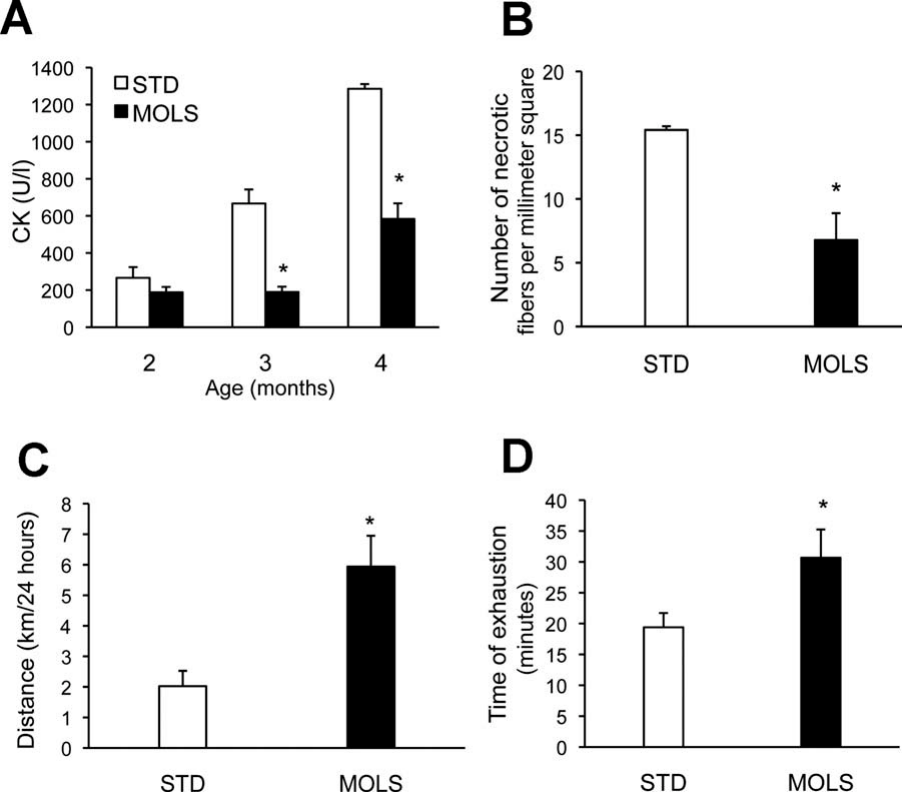
Nitric oxide induces functional recovery in dystrophic mice. CK plasma levels (**A**), number of necrotic fibers counted in hematoxylin and eosin sections of isolated *Tibialis Anterior* (**B**), free wheel running (**C**), and treadmill test results (**D**) measured in α-sarcoglycan null mice that received STD or treatment with MOLS. Data are expressed as mean ± SEM, *n* = 15, *, *p* ≤ .05 versus STD. Abbreviations: CK, creatine phosphokinase; MOLS, molsidomine; STD, standard diet.

To investigate whether molsidomine treatment led to a functional amelioration of the muscle, we measured the CK serum levels, the number of necrotic fibers in diaphragm muscle sections, and evaluated muscle functional recovery analyzing the voluntary movement and resistance to fatigue by the free wheel and treadmill tests, respectively. Molsidomine reduced CK serum levels (Fig. 6A) and preserved muscle integrity with reduced numbers of necrotic fibers in diaphragm muscle sections (Fig. 6B). In addition, molsidomine enhanced significantly spontaneous and forced motor activities indicating that not only morphological but also functional recovery of the muscle had taken place (Fig. 6C, 6D). Thus, treatment with molsidomine of α-SG null mice leads to a long-term reduction of muscle damage with functional recovery.

**Figure 7:**
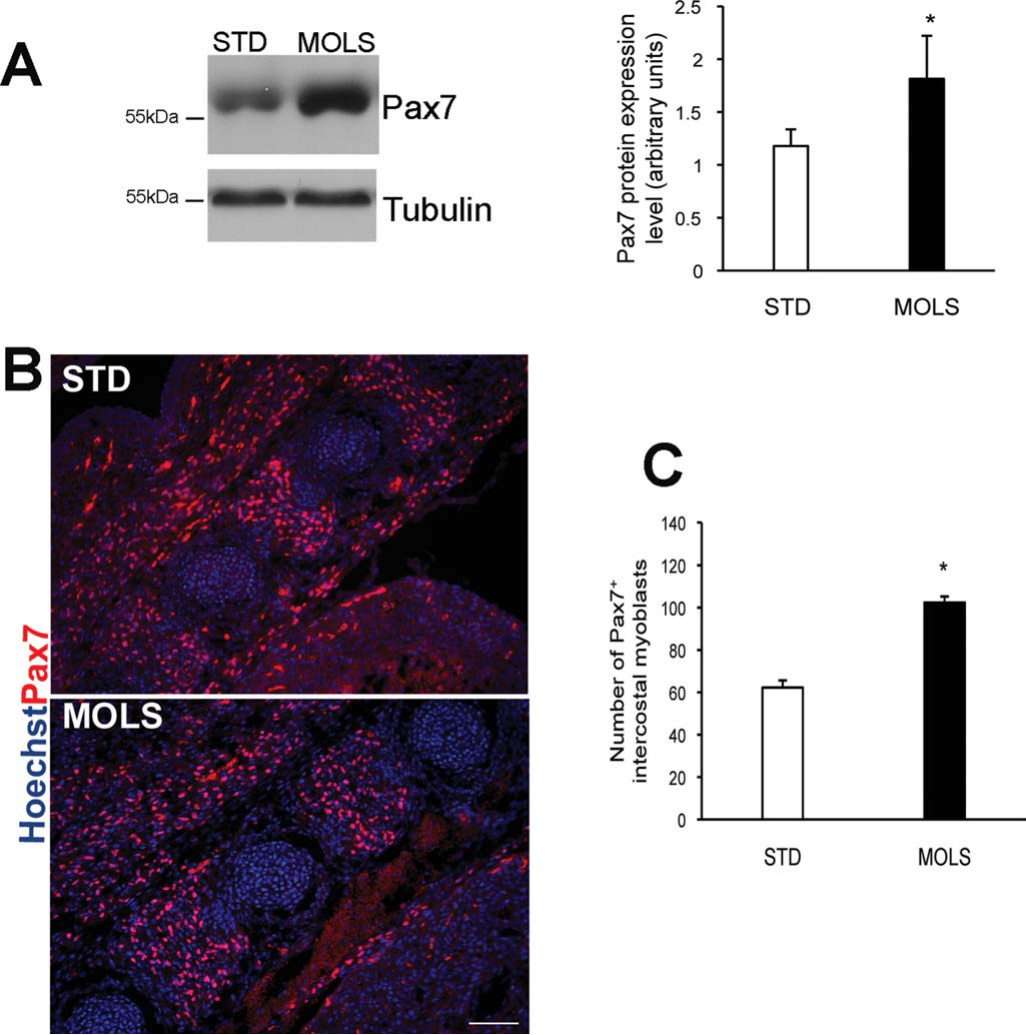
Nitric oxide reduces stem cell loss in dystrophic muscles during late embryogenesis. (**A**): Western blot analysis of Pax7 expression and of α-tubulin used as an internal control in homogenates of α-sarcoglycan null embryos from pregnant mothers receiving STD or treatment with MOLS. The left panel shows a representative Western blot, the right graph the Pax7 expression values, normalized against α-tubulin expression, *n* = 15, *, *p* ≤ .05 versus STD. (**B**): Representative images of Pax7 immunostaining of embryo intercostal sections obtained from the same groups of embryos. Pax7 staining is in red while nuclei are in blue. Scale bar = 75 μm. (**C**): Number of intercostal myoblasts expressing Pax7 counted in embryo intercostal sections obtained from the same groups of embryos. Data are expressed as mean ± SEM, *n* = 15, *, *p* ≤ .05 versus STD. Abbreviations: MOLS, molsidomine; STD, standard diet.

Stem cell loss in dystrophic muscles begins already during late embryogenesis [50]. We investigated if the beneficial effect of NO that we demonstrated in adult dystrophic mice was observed also during embryonic and fetal myogenesis. α-SG null female mice were treated with the molsidomine-enriched diet or with STD during pregnancy and embryos analyzed at day 17.5 of gestation. The expression of Pax7 in homogenates was evaluated by Western blotting and the number of Pax7^+^ cells in sections was counted after immunostaining as a measure of the total myogenic stem cells content. STD-treated α-SG null embryos displayed a reduced total Pax7 protein content with respect to the molsidomine-treated embryos (Fig. 7A). Moreover, analysis of sections demonstrated a recovery of Pax7^+^ cell loss in α-SG null NO-treated embryos (Fig. 7B, 7C). Thus molsidomine has beneficial effects also during embryonic myogenesis of α-SG null mice.

## DISCUSSION

In this study, we demonstrate that NO controls SC number and fate in such a way that it prevents the exhaustion of the reserve pool of these cells under conditions of severe muscle damage. We found that regulation by NO of SC number and fate is physiological, as inhibition or genetic ablation of nNOS in fibers is per se sufficient to induce a progressive reduction of the regenerative capacity of muscles. NO stimulated proliferation of SC via generation of cGMP and increased the number of Pax7^+^/Myf5^−^ SC via a cGMP-independent pathway. These actions appear coordinated to increase SC number per fiber.

The cGMP-dependent effect on proliferation is in line with previous reports about the mitogenic action of NO, for which the effectors downstream of cGMP have been identified [46, 47, 51]. The second mechanism, independent of cGMP, is instead new and found to require the noncanonical Wnt pathway and specifically Vangl2. NO enhanced the expression of Vangl2 while Vangl2 silencing completely abolished the action of NO. Thus, while we cannot exclude that other downstream effectors of NO participated in its effect on Pax7^+^/Myf5^−^ SC expansion, our results identify Vangl2 as necessary for this action of NO.

Vangl2 regulates planar cell polarity and the converged-extension movements in gastrulation and neurulation, as well as cell adhesion, motility, polarity, and gene transcription in a RhoA-dependent way; Vangl2 null mice do not survive past birth and show developmental defects in the nervous, cardiovascular, skeletal, and vestibular systems [52–54].

Despite its relevance in development, little is known on the functional role of Vangl2 and the noncanonical Wnt pathway in myogenesis, apart from recent evidence of its role in symmetric cell division and expansion of the quiescent SC pool, influencing the distribution and expression of members of the planar cell polarity pathway [25]. In addition, the noncanonical Wnt pathway plays a role in differential activation of genes in epaxial or hypaxial progenitors, via the action of protein kinase C (PKC) [55, 56]. The identification of an interplay between NO signaling and the Vangl2/noncanonical Wnt pathway in SC self-renewal, and the demonstration of the functional and therapeutic relevance of such an effect, extend the importance of this pathway in muscle and other tissues. The action of NO on noncanonical Wnt signaling was to date limited to its role in the control of cell division, morphogenesis, and cell movement/polarity during early Xenopus development [57], and in the regulation of c-Jun N-terminal kinase (JNK)/stress-activated protein kinase–related SC movements in fibers [58]. Several of the components of the noncanonical Wnt pathway, for example, RhoA, JNK, and PKC, have however already been shown to be regulated by NO in a variety of different cell systems, although a connection with this specific pathway was not established [59–61]. Thus the possibility that the Vangl2/noncanonical Wnt pathway participates to these other actions of NO should be investigated.

The observation that the Vangl2/noncanonical Wnt pathway is activated by NO and is involved in the NO-driven SC regulation and muscle repair also suggests that the role of NO in skeletal muscle is broader than previously envisaged. First, we demonstrate that the action of NO is crucial not only in preserving the pool of cells that sustain adult myogenesis but also during embryonic myogenesis. This might lead to a re-evaluation of the role of NO in early myogenesis, in which the role of this messenger has been poorly investigated to date. In addition, some of the actions through which NO acts to regulate adult muscle function are independent of cGMP but the precise effectors have not always been identified [33, 34, 62, 63]. Assessment of the involvement in these events of NO-dependent Wnt-signaling would lead to a better understanding of its contribution to skeletal muscle physiology.

The importance of these data goes beyond muscle physiology and has important consequences for the therapy of muscular dystrophy. The maintenance of the SC endogenous pool is crucial to the therapy of muscular dystrophy, a disease in which the continuous myofiber damage leads to repeated rounds of degeneration and repair that cause the progressive fall of SC regenerative ability [11].

To date an effective pharmacological strategy to muscular dystrophy is still lacking and the only available therapeutic protocols, based on corticosteroids are not resolutive and associated with severe side effects [64]. The recent approaches to muscular dystrophy therapy based on stem cell delivery [65, 66] and exon skipping [67, 68] are promising; however, they are expensive and in some cases are useful for only subsets of patients. Pharmacological strategies targeting key events downstream of the genetic defect can compensate in several genetic diseases, at least partially, the pathological consequence of the genetic defect and have therapeutic potential; in addition, they are cost effective and of broad applicability [69].

NO delivery was considered a possible therapeutic strategy based on the fact that mislocalization of nNOSμ and reduction of its activity due to dystrophin complex destruction contribute to damage progression [35–38], and that restoration of NO signaling by nNOS overexpression ameliorates muscle function [70, 71]. Indeed some NO donors were found to limit muscle damage and to gain therapeutic efficacy in combination therapies with non steroidal anti-inflammatory drugs [39, 49, 72] or corticosteroids [73].

The role of NO in myogenesis has been consequently intensely investigated and the messenger has been reported to promote activation, differentiation, and fusion of SC via various mechanisms [74–80]. In addition, NO is a vasodilator and angiogenic factor and also these effects conceivably contribute to the therapeutic action of NO in dystrophy. Indeed, increasing the vasculature in muscular dystrophy ameliorates the histological and functional phenotypes associated with this disease [81]. Nonetheless, none of these actions, while important, appear sufficient to explain the long-term efficacy of NO-based therapies. Although we have not clarified whether and by which cellular mechanism(s) NO increases cycling lifetime of SC, the NO-dependent increased proliferation of SC may allow an increased number of these cells to enter the self-renewal pathway, rather than into immediate myogenic differentiation. This mechanism may explain why NO is effective long-term in the chronic muscle degeneration observed in muscular dystrophy and further supports its therapeutic role.

A previous short-term study in the *mdx* mouse model of dystrophy showed that molsidomine reduces creatine kinase serum levels, muscle necrosis as well as the fatty and connective tissue infiltrates [82]. We now demonstrate that molsidomine is significantly effective long-term and per se, also on muscle function and recovery, and in such a severe model of dystrophy as the α-SG null mouse. This finding enhances the possibility of optimizing NO-based therapies. Why molsidomine is more effective than the other NO donors tested so far remains to be established. A likely possibility is that molsidomine is particularly effective in increasing SC number. Such an action may depend on the pharmacokinetic profile of NO release by molsidomine, or on the fact that it releases alongside NO also biologically active nitrites and nitrates [83]. These compounds appear to have beneficial functions in skeletal muscle [84, 85].

## CONCLUSION

Our results identify NO as a key messenger in SC fate regulation and expand the significance of the Vangl2-dependent Wnt noncanonical pathway in myogenesis. The demonstration that molsidomine prevents the decrease of SC under pathological conditions with long-term functional improvement of muscle function reinforces the notion that optimizing NO-based strategies for muscular dystrophy is a valuable pathways to pursue in therapeutic perspective.

## Supporting Information

Supplemental Figure 1 (A) Representative images of EDL fibres cultured for 36/42 or 96 h in floating conditions: Pax7 staining is in green while nuclei are stained in blue with Hoechst. Scale bar = 25 μm. (B) Representative images of fibres after 96 h of culture. The upper panels show the staining for nuclei (blue), Myf5 (red) or MyoD (green). The lower panels show the staining for nuclei (blue) Myf5 (red) and Myogenin (green). Scale bar = 75 μm. (C) Number of Pax7+/Myf5- cells in single fibres cultured in floating conditions in the presence of increasing concentrations of SIN-1 (left panel) or L-NAME (right panel). Data are expressed as mean ± s.e.m. and normalised on fibre nuclei number. At least 50 fibres were analysed in each experiment, n = 3, asterisk: p ≤ 0.05. (D) Number of Pax7+/Myf5- (left) or Pax7−/Myf5+ (right) cells in single fibres cultured in floating conditions in the presence of the NO donor DETA-NO (10μM) or vehicle (NT). Data are expressed as mean ± s.e.m. and normalised on fibre nuclei number. At least 50 fibres were analysed, n = 3, asterisk: p ≤ 0.05.

Supplemental Figure 2 (A) Representative images of TA sections isolated before (0 days) or 5 and 10 days after CTX damage and stained for nuclei (Hoechst, blue), Pax7 (green) and Laminin (red). Scale bar = 75 μm. (B) Representative images of H&E sections of TA isolated before (0 days) or 5 and 10 days after CTX damage. Scale bar = 100 μm. (C) Number of Pax7−/Myf5+ cells counted in single fibres isolated from EDL after multiple injections of CTX. Fibres were obtained after CTX treatment from nNOS- /- mice or from wild type mice that received standard diet (STD) or a diet containing molsidomine (MOLS) or with L-NAME in drinking water. Data are expressed as mean ± s.e.m. (≥ 100 fibres for each experiment, n = 3) and normalised on fibre nuclei number; asterisks: p ≤ 0.05 vs. STD. (D) Real Time PCR analysis of Myf5 expression in myofibres isolated after CTX injection. Results are expressed as fold changes over the values in STD set arbitrarily at 1, n = 3, asterisk: p ≤ 0.05 vs. STD. (E) Semiquantitative PCR analysis of Pax7 expression in flow cytometry-sorted cells (G0 vs G2/M fractions). Results are expressed as fold changes over the values in the G2/M fraction set arbitrarily at 1, n = 3, asterisk: p ≤ 0.05 vs. G2/M. (F) Representative dot plots of isolated myoblasts obtained from wt animals treated with STD (b), molsidomine (c) or L-NAME (d), or from nNOS−/- mice (e); a) shows a reference sample stained with Hoechst alone and used to distinguish between the G0 and G1 phases. The gates employed for analysis of the G0 and G2/M phases are shown in b. (G) Percentage of cells in the G0 phase measured in myoblast progenitor cells isolated from leg muscles of mice after CTX injection, n = 3, asterisks: p ≤ 0.05 vs. STD.

Supplemental Figure 3 (A) Real Time PCR analysis of Frzd7 (FZD7), β catenin (CTNNB1) and Wnt7a (WNT7a) expression in single fibres cultured in floating conditions and obtained from EDL of wild type mice treated with SIN-1, L-NAME or vehicle (NT), or obtained from nNOS−/- mice. Results are expressed as fold changes over the values in NT set arbitrarily at 1, n = 4, asterisks: p ≤ 0.05 vs. NT. (B) Silencing efficiency of the Vangl2-specific siRNA determined by Real Time PCR analysis in single fibres transfected with the specific siRNAs or scramble. Results are expressed as fold changes over the values in NT set arbitrarily at 1, n = 3, asterisk: p ≤ 0.05 vs. NT. (C) Real Time PCR analysis of FZD7, CTNNB1 and WNT7a mRNA expression in single myofibres isolated after CTX injection. Fibres were obtained after CTX injection of wild type mice that received standard diet (STD) or a diet containing molsidomine (MOLS) or L-NAME in drinking water. nNOS−/- mice were analysed in parallel. Results are expressed as fold changes over the values in STD set arbitrarily at 1, n = 3, asterisk: p ≤ 0.05 vs. STD.

Supplemental Figure 4 Number of Pax7−/Myf5+ cells (A), Real Time PCR analysis of Myf5 expression (B) or Frzd7 (FZD7), β-catenin (CTNNB1) and Wnt7a (WNT7a) (C) in single fibres isolated from 9 months old a-SG null mice that received standard diet (STD) or the diet containing molsidomine (MOLS). Results in all panels are expressed as fold changes over the values in STD set arbitrarily at 1 (n = 3), asterisks: p ≤ 0.05 vs. STD.
