# Supplementary figures and images for: Nitric Oxide Sustains Long-Term Skeletal Muscle Regeneration by Regulating Fate of Satellite Cells Via Signaling Pathways Requiring Vangl2 and Cyclic GMP
Source: Stem Cells. 2011 Nov 14;30(2):197–209. doi: 10.1002/stem.783 (PMC3378700; doi:10.1002/stem.783)

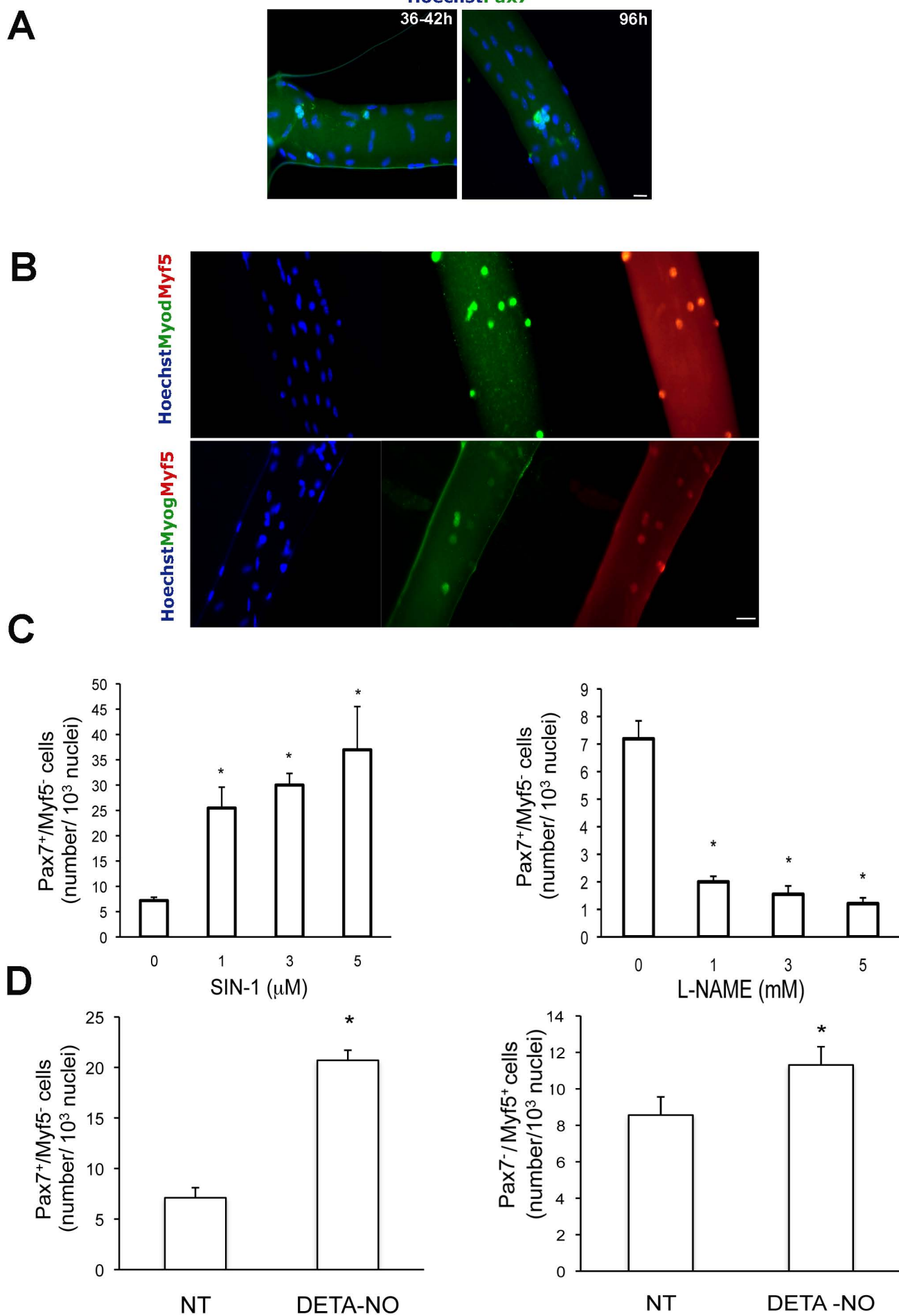

Supplement: Supplemental Figure 1 — (A) Representative images of EDL fibres cultured for 36/42 or 96 h in floating conditions: Pax7 staining is in green while nuclei are stained in blue with Hoechst. Scale bar = 25 μm. (B) Representative images of fibres after 96 h of culture. The upper panels show the staining for nuclei (blue), Myf5 (red) or MyoD (green). The lower panels show the staining for nuclei (blue) Myf5 (red) and Myogenin (green). Scale bar = 75 μm. (C) Number of Pax7+/Myf5- cells in single fibres cultured in floating conditions in the presence of increasing concentrations of SIN-1 (left panel) or L-NAME (right panel). Data are expressed as mean ± s.e.m. and normalised on fibre nuclei number. At least 50 fibres were analysed in each experiment, n = 3, asterisk: p ≤ 0.05. (D) Number of Pax7+/Myf5- (left) or Pax7−/Myf5+ (right) cells in single fibres cultured in floating conditions in the presence of the NO donor DETA-NO (10μM) or vehicle (NT). Data are expressed as mean ± s.e.m. and normalised on fibre nuclei number. At least 50 fibres were analysed, n = 3, asterisk: p ≤ 0.05. [file stem0030-0197-SD1.pdf]

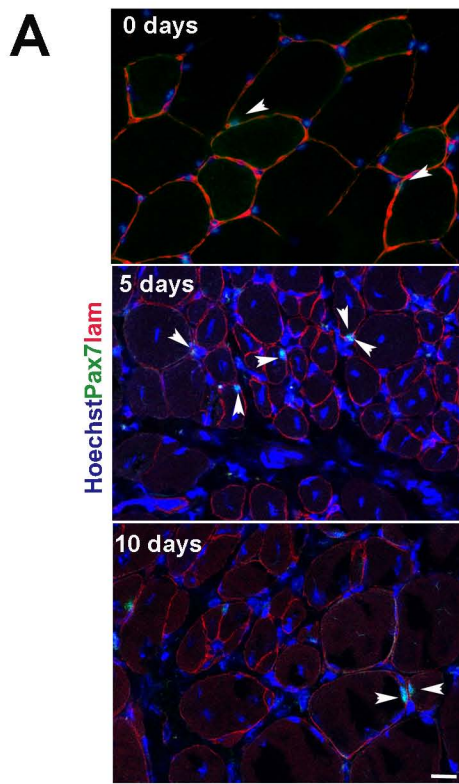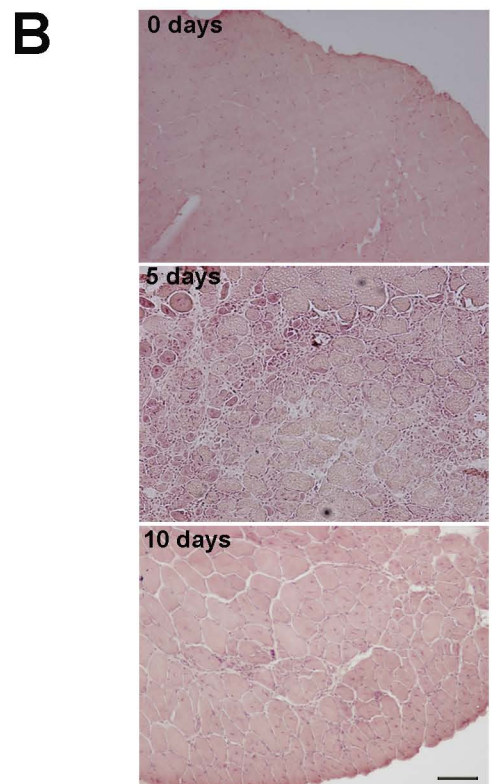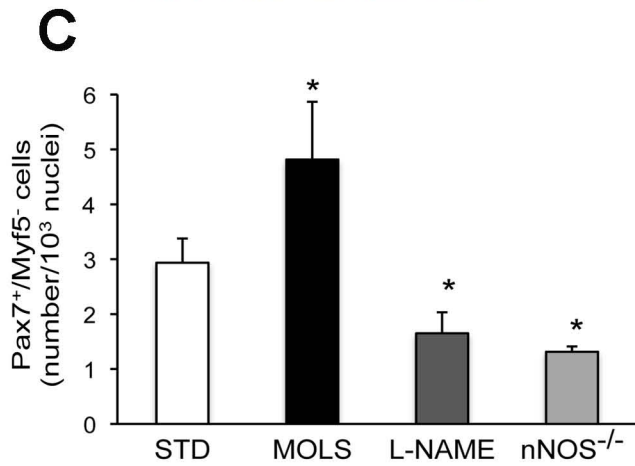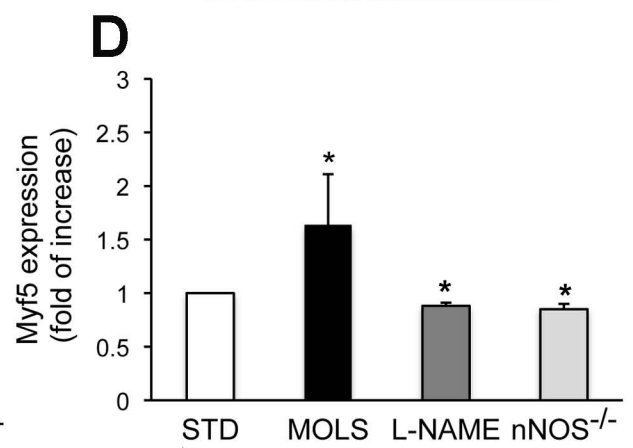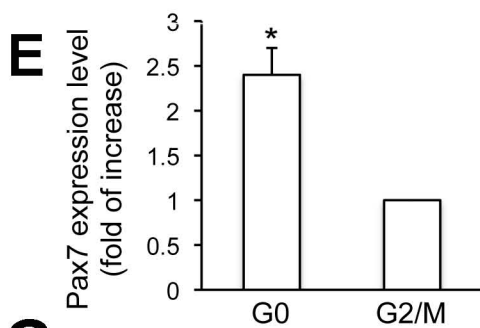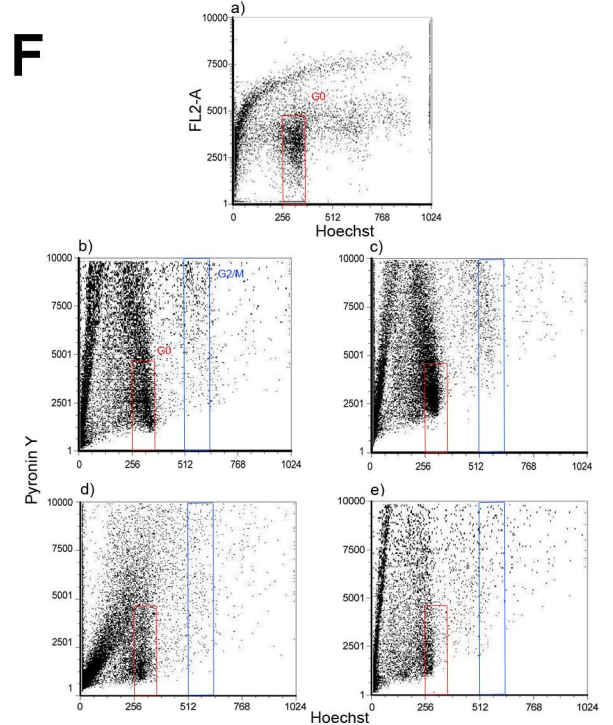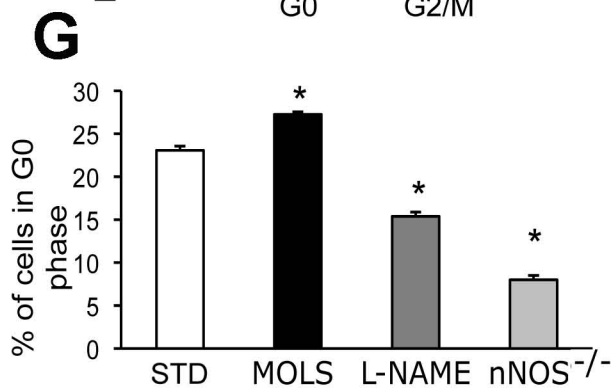

Supplement: Supplemental Figure 2 — (A) Representative images of TA sections isolated before (0 days) or 5 and 10 days after CTX damage and stained for nuclei (Hoechst, blue), Pax7 (green) and Laminin (red). Scale bar = 75 μm. (B) Representative images of H&E sections of TA isolated before (0 days) or 5 and 10 days after CTX damage. Scale bar = 100 μm. (C) Number of Pax7−/Myf5+ cells counted in single fibres isolated from EDL after multiple injections of CTX. Fibres were obtained after CTX treatment from nNOS- /- mice or from wild type mice that received standard diet (STD) or a diet containing molsidomine (MOLS) or with L-NAME in drinking water. Data are expressed as mean ± s.e.m. (≥ 100 fibres for each experiment, n = 3) and normalised on fibre nuclei number; asterisks: p ≤ 0.05 vs. STD. (D) Real Time PCR analysis of Myf5 expression in myofibres isolated after CTX injection. Results are expressed as fold changes over the values in STD set arbitrarily at 1, n = 3, asterisk: p ≤ 0.05 vs. STD. (E) Semiquantitative PCR analysis of Pax7 expression in flow cytometry-sorted cells (G0 vs G2/M fractions). Results are expressed as fold changes over the values in the G2/M fraction set arbitrarily at 1, n = 3, asterisk: p ≤ 0.05 vs. G2/M. (F) Representative dot plots of isolated myoblasts obtained from wt animals treated with STD (b), molsidomine (c) or L-NAME (d), or from nNOS−/- mice (e); a) shows a reference sample stained with Hoechst alone and used to distinguish between the G0 and G1 phases. The gates employed for analysis of the G0 and G2/M phases are shown in b. (G) Percentage of cells in the G0 phase measured in myoblast progenitor cells isolated from leg muscles of mice after CTX injection, n = 3, asterisks: p ≤ 0.05 vs. STD. [file stem0030-0197-SD2.pdf]

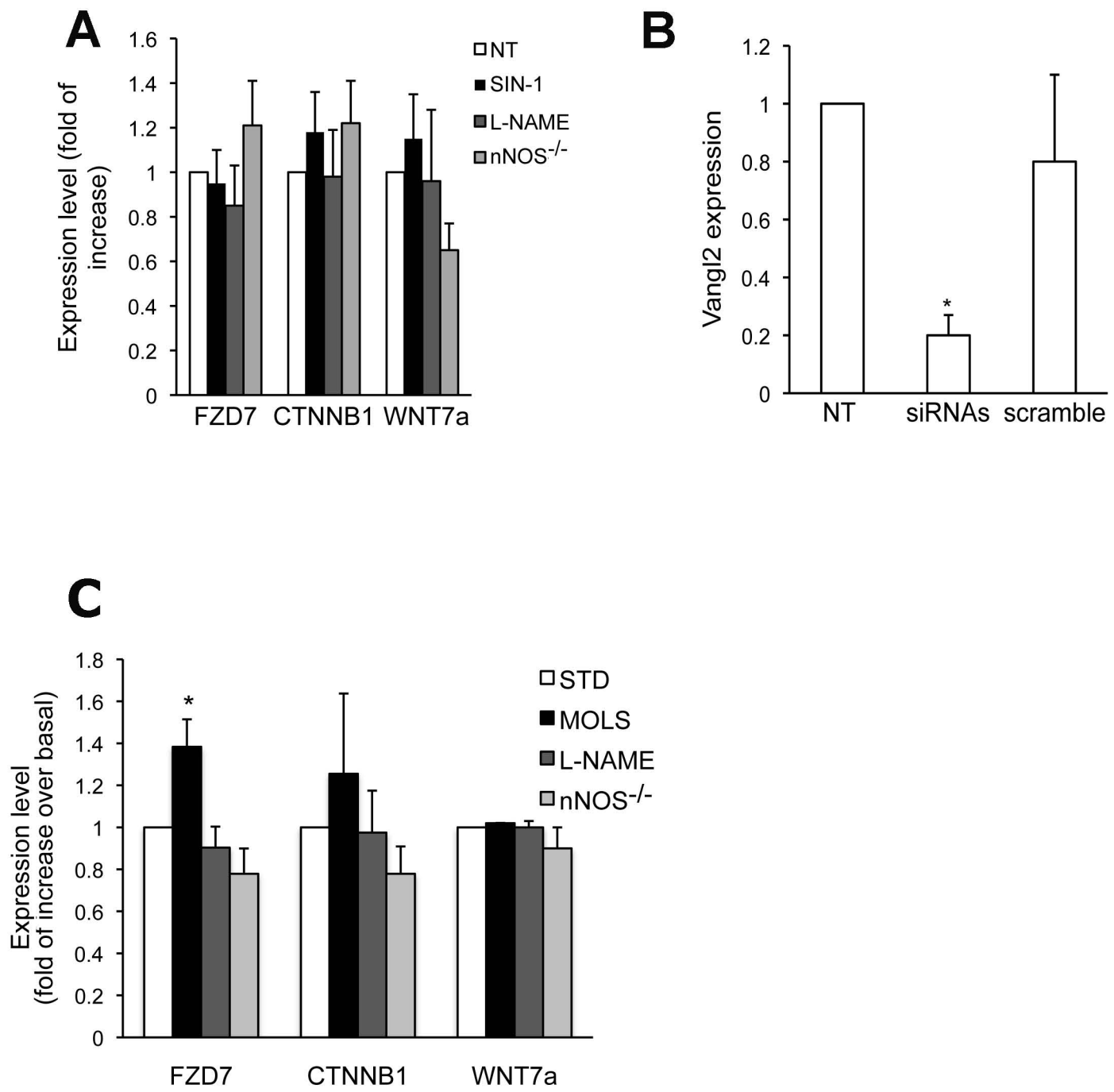

Buono et al supplementary Fig. 3

Supplement: Supplemental Figure 3 — (A) Real Time PCR analysis of Frzd7 (FZD7), β catenin (CTNNB1) and Wnt7a (WNT7a) expression in single fibres cultured in floating conditions and obtained from EDL of wild type mice treated with SIN-1, L-NAME or vehicle (NT), or obtained from nNOS−/- mice. Results are expressed as fold changes over the values in NT set arbitrarily at 1, n = 4, asterisks: p ≤ 0.05 vs. NT. (B) Silencing efficiency of the Vangl2-specific siRNA determined by Real Time PCR analysis in single fibres transfected with the specific siRNAs or scramble. Results are expressed as fold changes over the values in NT set arbitrarily at 1, n = 3, asterisk: p ≤ 0.05 vs. NT. (C) Real Time PCR analysis of FZD7, CTNNB1 and WNT7a mRNA expression in single myofibres isolated after CTX injection. Fibres were obtained after CTX injection of wild type mice that received standard diet (STD) or a diet containing molsidomine (MOLS) or L-NAME in drinking water. nNOS−/- mice were analysed in parallel. Results are expressed as fold changes over the values in STD set arbitrarily at 1, n = 3, asterisk: p ≤ 0.05 vs. STD. [file stem0030-0197-SD3.pdf]

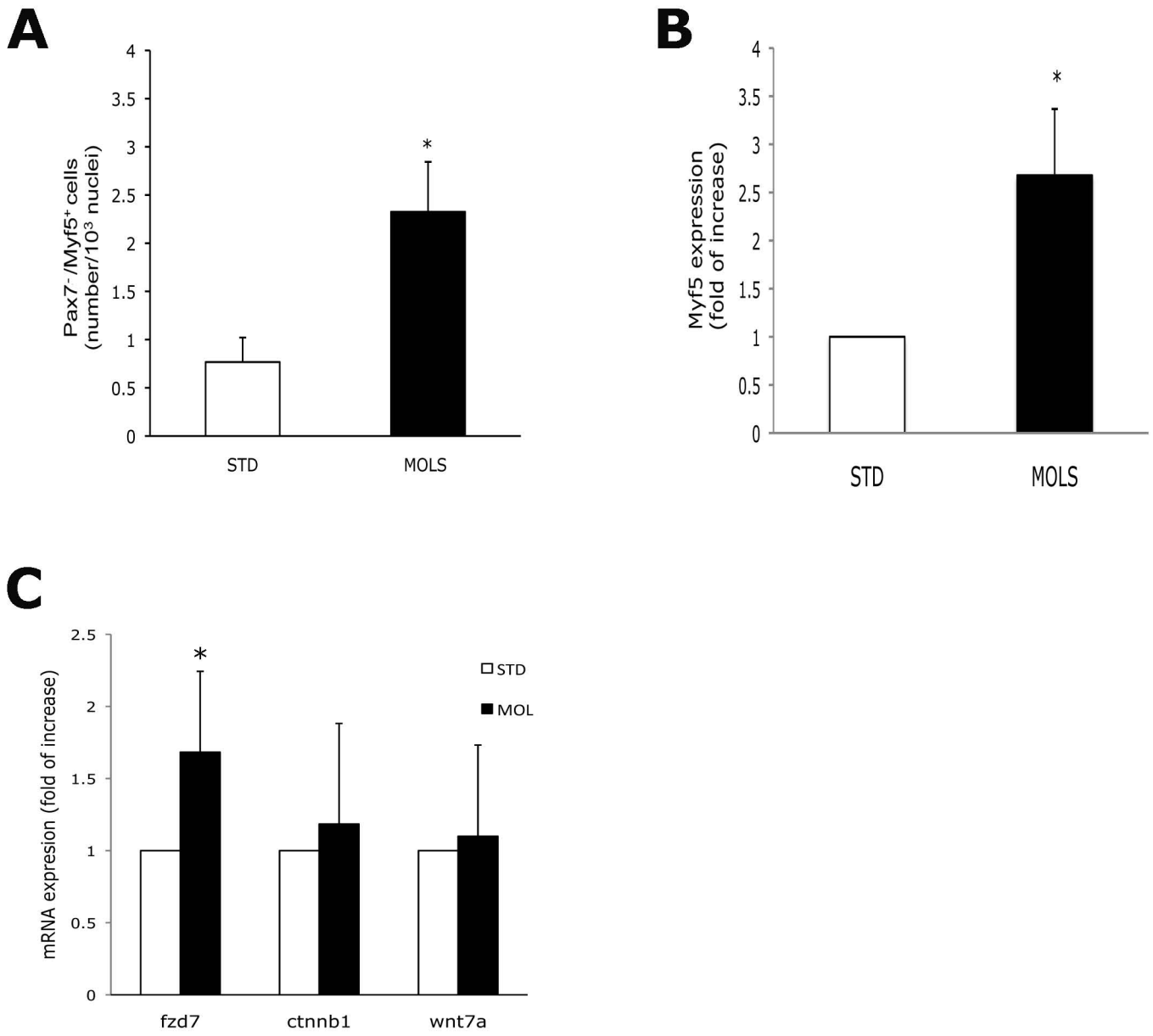

Buono et al. Supplementary fig.4

Supplement: Supplemental Figure 4 — Number of Pax7−/Myf5+ cells (A), Real Time PCR analysis of Myf5 expression (B) or Frzd7 (FZD7), β-catenin (CTNNB1) and Wnt7a (WNT7a) (C) in single fibres isolated from 9 months old a-SG null mice that received standard diet (STD) or the diet containing molsidomine (MOLS). Results in all panels are expressed as fold changes over the values in STD set arbitrarily at 1 (n = 3), asterisks: p ≤ 0.05 vs. STD. [file stem0030-0197-SD4.pdf]
