# Supplementary material for: Nitric Oxide Sustains Long-Term Skeletal Muscle Regeneration by Regulating Fate of Satellite Cells Via Signaling Pathways Requiring Vangl2 and Cyclic GMP
Source: Stem Cells. 2011 Nov 14;30(2):197–209. doi: 10.1002/stem.783 (PMC3378700; doi:10.1002/stem.783)
Supplement: Supplementary file 5 [file stem0030-0197-SD5.pdf]

SUPPLEMENTAL MATERIAL

SOURCES OF ANIMALS AND MATERIALS

Animals

α-SG null mice were a kind gift of Dr. K Campbell, Iowa University, IA, USA; 129S2/SvpasClr and C57BL/6J wild type mice were from Charles River, Wilmington, MA USA; nNOS null mice (B6.129S4-Nos1tm1Plh/J) were from Jackson Laboratories, Bar Harbor, Maine, USA.

Materials

Antibodies sources and dilutions were as follows: anti Myf5, a rabbit polyclonal antibody from Santa Cruz Biotechnology, Inc., Santa Cruz, CA, USA, was used at a 1:200 dilution; anti MyoD, a rabbit polyclonal antibody from Santa Cruz Biotechnology, Inc., was used at a 1:250 dilution; anti Pax7, a mouse monoclonal antibody from Developmental Studies Hybridoma Bank Iowa City, Iowa USA, was used at a 1:2 dilution; anti Myogenin, a mouse monoclonal antibody from Developmental Studies Hybridoma Bank, was used at a 1:2 dilution; anti-α tubulin, a rabbit antibody from Abcam, Cambridge, UK, was used at a 1:1000 dilution; anti-Laminin, a monoclonal antibody from Sigma was used at a 1:300 dilution; anti-Vangl2, a monoclonal antibody from R&D System, Minneapolis, MN, USA, was used at a 1:200 dilution. Secondary antibodies were AlexaFluor 488, 546 or 647 from Invitrogen Life Science, CA, USA, or horseradish peroxidase secondary antibodies from Biorad, Hercules, CA, USA, all used at a 1:1000 dilution.

SIN-1 and DETA-NO were from Enzo Life Sciences, New York, NY, USA; Mastecycler 5330 was from Epperdorf; Wnt7a was from R&D System; goat serum was from Vector Laboratories Inc., Burlingame, CA, USA; the Prolong® Gold antifade reagent, Lipofectamine 2000 and Trizol reagents were from Invitrogen.

The ABI PRISM® 7900HT Fast Real-Time PCR Systems, its specific gene expression assays, the siRNAs and scramble sequences were purchased from Applied Biosystems, Foster City, CA, USA; The enhanced chemiluminescence was from GE Healtcare, Buckingamshire, UK. The Vantage DiVa Sorter was from Becton Dikinson; QuantiTect Reverse Transcription kit from Qiagen, Germantown, MD, USA.

Molsidomine, L-NAME, CTX, BrdU, 8Br-cGMP, Hoechst 33342, Pyronin Y, JumptStart, redaccuTaq and all other reagents were from Sigma-Aldrich.

Microscopes and objective lenses

Images of immunofluorescence studies on single fibres were acquired using a Leica CTR600 microscope (Leica, Mannheim, Germany), equipped with a DFC350FXR2-075951105 camera. Images were taken using the NPLAN L20.0x0.40 DRY (0.40 numeric aperture 1x magnification) or the HCXPL FLUORTARL 40.0x0.60 DRY (0.60 numeric aperture 1x magnification) objective lenses, using the LAS/AF acquisition programme (Leica). Linear adjustments of images were obtained using Adobe Photoshop CS4. The number of cells or nuclei was measured using a digitised imaging system (ImageJ 1.38 x National Institute of Health).

Images from histology studies on tissue sections were acquired with a Nikon E600 microscope (Nikon, NY USA) and a Nikon DXM 1200 camera, using ATC-1 (Nikon) as the acquisition programme and the objective lens PLAFLUOR L10.0x0.30 DRY

(0.30 numeric aperture 1x magnification). Images from immunoflurescence studies on tissue sections were acquired with a Laser scanning confocal (TCS SP2. Leica) and a HCX PL APO CS 40.0x1.25 OIL UV objective lens (numerical aperture 1.250000 1x magnification). In the case of Pax7 staining after CTX damage, only regenerated areas were measured to avoid detection of unspecific signals due to diffuse necrosis of fibres. Images from embryo sections were acquired using the Leica CTR600 microscope equipped with the NPLAN L20.0x0.40 DRY (0.40 numeric aperture 1x magnification) objective lens. Linear adjustments of images were done using Adobe Photoshop CS4. Ten random images for each muscle or embryo were taken. The number of cells, fibres or nuclei was measured using a digitised imaging system (ImageJ 1.38 x National Institute of Health). The number of $Pax7^+$ or $Myf5^+$ cells was normalised to the total number of nuclei in single fibres and expressed as number/10^3 nuclei.

SUPPLEMENTAL METHODS

Cell Cycle Analysis and Sorting

Myogenic precursor cells were isolated from leg muscles[44] and stained with 10μM of Hoechst 33342 and 2.5 μg/ml of Pyronin Y for 45 min at 37°C[19]. Cells were analysed and sorted on the basis of Hoechst and Pyronin fluorescence as G0 and G2/M cell-cycle fractions by the Vantage DiVa Sorter and expressed as percentage of cells after debris subtraction. The gate to distinguish G0 from G1 phase in the dot plot double staining analysis was chosen based on a reference sample stained with Hoechst alone; G0, S or G2/M phases were distinguished based on the DNA content in linear mode

acquisition. Expression of Pax7 and Myf5 in cells of G0 and G2/M fractions was tested by semi-quantitative PCR using the same amount of RNA (1μg of RNA/sample) and employing the following primers:

Pax7-F GGTTGTGTCTCCAAGATTCTG

Pax7-R GTAGTGGGTCCTCTCGAAG

β-Actin-F GTCAGAAGGACTCCTACGTG

β-Actin-R GAGTACTTGCGCTCAGGGG

The cDNA extracts (prepared as described above) were amplified with JumptStart redaccuTaq and Mastecycler 5330 using the following conditions: 96 °C for 30 s, 35 cycles of denaturation at 94 °C for 15 s, annealing at 60 °C for 30 s, extension at 68 °C for 30 s, and final extension at 68 °C for 6 min. The PCR products were analysed by 2% agarose gel electrophoresis and stained with ethidium bromide. Calculation of relative expression was normalised using β-Actin. Results shown are averages of three reproducible independent experiments.
